# Time course of skeletal muscle regeneration after severe trauma: Muscle function against the background of MRI and histological findings

**DOI:** 10.3109/17453674.2010.539498

**Published:** 2011-02-10

**Authors:** Tobias Winkler, Philipp von Roth, Georg Matziolis, Maria R Schumann, Sebastian Hahn, Patrick Strube, Gisela Stoltenburg-Didinger, Carsten Perka, Georg N Duda, Stephan V Tohtz

**Affiliations:** ^1^Center for Musculoskeletal Surgery and Julius Wolff Institute Berlin, Brandenburg Center for Regenerative Therapies; ^2^Institute of Neuropathology, Charité – Universitaetsmedizin Berlin, Berlin, Germany

## Abstract

**Background and purpose:**

Animal models of skeletal muscle injury should be thoroughly described and should mimic the clinical situation. We established a model of a critical size crush injury of the soleus muscle in rats. The aim was to describe the time course of skeletal muscle regeneration using mechanical, histological, and magnetic resonance (MR) tomographic methods.

**Methods:**

Left soleus muscles of 36 Sprague-Dawley rats were crushed in situ in a standardized manner. We scanned the lower legs of 6 animals by 7-tesla MR one week, 4 weeks, and 8 weeks after trauma. Regeneration was evaluated at these times by in vivo measurement of muscle contraction forces after fast-twitch and tetanic stimulation (groups 1W, 4W, 8W; 6 per group). Histological and immunohistological analysis was performed and the amount of fibrosis within the injured muscles was determined histomorphologically.

**Results:**

MR signals of the traumatized soleus muscles showed a clear time course concerning microstructure and T1 and T2 signal intensity. Newly developed neural endplates and myotendinous junctions could be seen in the injured zones of the soleus. Tetanic force increased continuously, starting at 23% (SD 4) of the control side (p < 0.001) 1 week after trauma and recovering to 55% (SD 23) after 8 weeks. Fibrotic tissue occupied 40% (SD 4) of the traumatized muscles after the first week, decreased to approximately 25% after 4 weeks, and remained at this value until 8 weeks.

**Interpretation:**

At both the functional level and the morphological level, skeletal muscle regeneration follows a distinct time course. Our trauma model allows investigation of muscle regeneration after a standardized injury to muscle fibers.

Injuries of skeletal muscle tissue cause deficiencies in local muscle function that can also affect bone healing (Duda et al. 2003, Schaser et al. 2003, Utvag et al. 2003, Harry et al. 2008) or the long-term success of a prosthesis (Kleemann et al 2003, Perka et al. 2005).

Despite the fact that there have been several experimental approaches, no methods for the treatment of the main causes of these deficiencies—i.e. the loss of contractile muscle substance and the formation of fibrosis—have been taken into routine clinical use. One reason is that the results of putative therapies are often only described mono-dimensionally, in most cases histologically, and rarely functionally or with in vivo diagnostic methods.

Animal models of muscle contusion injuries should closely mimic the clinical situation. Among them, open crush injuries allow standardized evaluation of regeneration in a selected muscle (Schultz et al. 1985, Rushton et al. 1997). For the execution of the trauma, either forceps (McGeachie and Grounds 1987, Kurek et al. 1997, Fink et al. 2003) or custom-made devices have been used (Jarvinen and Sorvari 1975, Rushton et al. 1997, Bunn et al. 2004).

Previous muscle-crush models have had the disadvantage of either affecting only part of the muscle or of impairing the organ innervation and blood supply. Two types can be found in the literature: the segmental crush (Schmalbruch 1976) and the complete crush, where only 4–6% of the muscle fibers remain intact (Fink et al. 2003). In the latter, myoneuronal junctions are damaged, which triggers not only regeneration of muscle substance but also initial innervation deficits. These deficits always lead to impaired healing (Saunders and Sissons 1953, Koishi et al. 1995, Dedkov et al. 2001, Pereira et al. 2006).

Histological analysis of the regenerative process after crush injury has been done by a number of authors, describing the initial phase of inflammation followed by satellite cell activation, myotube regeneration, and fibrosis of the muscle (Jarvinen and Sorvari 1975, Schmalbruch 1976, Sorokin et al. 2000, Bunn et al. 2004). It has been shown that the development of fibrotic tissue is one of the key factors for regenerative deficits of muscle function after trauma (Anderson et al. 1999, Nozaki et al. 2008). The development of intramuscular fibrosis over time after crush trauma has not, however, been quantitatively described.

Magnetic resonance imaging (MRI) can be used in the clinical evaluation of skeletal muscle injuries. Not only are T1- and T2- weighted spin echo sequences highly sensitive for detection of edema and bleeding in anatomical relation to the structures affected (Elsayes et al. 2006), but MR scans also allow the determination of intra-individual time courses after trauma.

The evaluation of muscle regeneration by contraction force measurements provides information about the functional outcome of therapeutic approaches (Sato et al. 2003, Negishi et al. 2005, Winkler et al. 2009). The description of a muscle trauma model should therefore not solely include morphological but also mechanical data.

One aim of our study was to establish a standardized model of selective trauma of the muscle substance of a single muscle, leaving the blood and nerve supply undamaged. This was achieved by developing a global crush injury of the rat soleus muscle with respect to the topographic arrangement of the innervating endplate zone. We also wanted to describe the regeneration of muscle contraction forces against the background of morphological changes within the muscle tissue following a critical size of injury in a skeletal muscle.

## Material and methods

### Animals

36 male Sprague Dawley rats weighing 450–550g were used for the study. The rats were housed at a constant temperature of 25°C with free access to pellet food and water. All animal experiments were carried out according to the policies and principles established by the Animal Welfare Act, the NIH Guide for Care and Use of Laboratory Animals, and the German national animal welfare guidelines. The study was approved by the local institute of health.

### Experimental procedure

All animals received an open crush trauma of the left soleus muscles. 1, 4, and 8 weeks after injury, muscle contraction forces were measured in vivo and the animals were killed (groups 1W, 4W, and 8W; n = 6 per group). The soleus muscles of both sides were harvested for histological evaluation. Soleus muscles of 4 animals were harvested on days 1, 2, and 4 after trauma for histological evaluation of the first days after injury. 6 animals were examined by sequential MRI measurements at 1, 4, and 8 weeks (group MRI, n = 6).

### Muscle trauma

**Figure 1.**
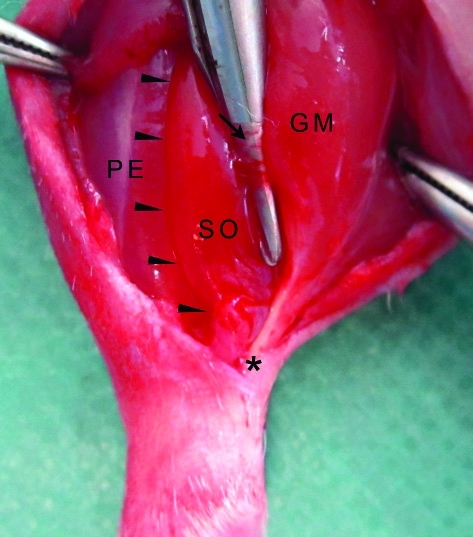
Innervation of soleus muscle and vascular supply. Arrowheads: lateral border of soleus muscle (SO); arrow: neurovascular bundle coming from the gastrocnemius muscle (GM) and entering at the medial border of the SO, the tip of the arrow indicating the soleus nerve; PE: peroneal muscles. The asterisk shows the Achilles tendon.

The rats were anesthetized by subcutaneous injections of 0.15 mL ketamine (100 mg/mL) and 0.15 mL xylazine (2%) diluted with 0.2 mL 0.9% saline. The left leg was shaved and disinfected with povidone-iodine. Through a 2-cm posterolateral longitudinal skin incision from the lateral gastrocnemius head to the Achilles tendon, the soleus muscle was mobilized and the soleus artery, vein and nerve, which arise from the mid-part of the gastrocnemius, were located. The medial border of the soleus muscle was dissected cranially and caudally to the neurovascular structures. A curved artery forceps, the jaws of which were protected by polyethylene tubes to avoid lesions of the muscle fascia, was introduced in direct proximity to the Achilles tendon and closed for 20 seconds. This procedure was repeated proximally 3 times, always in direct continuity to the respective distal crush. The insertion of the neurovascular bundle and a trapezoid region with a 3-mm long base at the insertion of the bundle and a 2-mm short base at the lateral margin of the soleus muscle was spared and 3 crushes were exerted proximal to this area in the manner described above (Figure 1). Standardized pressure on the muscle was ensured by closing the forceps to its third stage at each crush. The closing pressure at this stage corresponds to 112 (SD 5.1) N (data obtained from preliminary tests with the material testing device Zwick 1455 (Zwick GmbH, Ulm, Germany). After multiple irrigations, the superficial muscle and skin were closed.

### Measurement of muscle force

The animals were anesthetized as described before and received bilateral surgery. The sciatic nerve and the soleus muscle were exposed, sparing all neurovascular structures. The Achilles tendon was cut and the lower extremity was connected to a muscle force measuring device (Experimentria, Budapest, Hungary). The distal part of the soleus muscle was connected to a force transducer via a suture (4-0, silk). The sciatic nerve was subsequently stimulated with 9 mA/75 Hz bipolar pulses 5 times, for 0.1 second each (8 periods) with 5-second intervals between the pulses (Broniatowski et al. 1992, Silverman and Brull 1993). After this short twitch stimulation, the maximal muscle strength was measured using a protocol of 9 mA/75 Hz pulses 5 times, for 3 seconds each at 5-second intervals, reaching tetany in all cases. After completion of the muscle strength measurements, the animals were killed with an overdose of anesthetic and the soleus muscles were fixed in buffered paraformaldehyde (4%) for histological examination.

### MRI

6 animals were examined by MRI one, 4, and 8 weeks after muscle injury. Measurements were taken with a 7-tesla MRI spectrometer for small animals (Pharmascan 70/16; Bruker, Ettlingen, Germany). Images were processed with ParaVision software (Bruker BioSpin).

Rats were anesthetized by isofluran/O_2_ inhalation and were introduced into the spectrometer in a special animal handling system equipped with a heating pad and continuous monitoring (ECG and respiratory frequency). A rat brain coil with an inner diameter of 38 mm was used for transmission and reception. Each measurement involved one lower limb of the animal from the tibial plateau to the ankle joint. T1 (TR/TE: 1000/10.6 ms; scan time 12 min) and T2-turbo-rare sequences (TR/TE: 6351.6/75.6 ms; scan time 12 min) were performed with a resolution of 176 × 176 μm and a slice thickness of 500 μm.

Analysis of grayscale values was performed with ImageJ version 1.38i (National Institutes of Health, Bethesda, MD). The signal intensities of the voxels of the injured soleus muscles were quantified in the T1- and T2-weighted scans and compared to those of an uninjured reference muscle (anterior tibial muscle). The differences between the mean grayscale values of the soleus muscles were calculated for each time point (Figure 4).

### Histology

Hematoxylin and eosin (HE) staining and Picro-sirius red staining were used for descriptive analysis of the trauma. The stain Picro-sirius red selectively highlights collagenous connective tissues with a well-defined contrast. This stain was therefore used for quantitative analysis of fibrosis in the traumatized muscles, as previously described (Sweat et al. 1964, Wake et al. 2005). Furthermore, muscles were stained for nestin to localize the positioning of myotendinous junctions (MTJs) before and after trauma (Aarimaa et al. 2004) and with a-bungarotoxin to visualize the distribution of neuromuscular junctions within the soleus muscle. The endothelial marker factor VIII was used for the detection of vessels.

Soleus muscles were either paraformaldehyde-fixed, dehydrated, and embedded in paraffin or fresh-frozen in liquid nitrogen-cooled 2-methylbutan and embedded in Tissue Tek. The specimens were sectioned longitudinally (4 and 10 μm). For evaluation of connective tissue, sections were incubated for 60 min in Sirius red solution (5g Sirius red dissolved in 500 mL saturated picric acid). Differentiation was achieved by 2 washes with diluted acetic acid followed by a short dehydration in a series of graded alcohols.

The following antisera and antibodies were used for immunohistochemistry: mouse monoclonal antibody to rat nestin (1:800) (BD Biosciences Pharmingen, Franklin Lakes, NJ) and mouse monoclonal antibody to factor VIII (1:400) (Daco Cytomation, Glostrup, Denmark).

For light microscopy, the primary antibodies were visualized using appropriate avidin–biotin–peroxidase kits (Vector Laboratories, Burlingame, CA) and counterstained with hematoxylin. For fluorescence microscopy, Alexa Fluor 546-conjugated goat anti-mouse antibody (1:300) (Invitrogen Corporation, Camarillo, CA) was used as secondary antibody. DAPI was used as counterstain for all fluorescence stains. For endplate staining, the frozen sections were incubated for 2 h at room temperature with Alexa Fluor 488-conjugated a-bungarotoxin (2 μg/mL) (Invitrogen).

A blinded investigator evaluated the amount of collagenous connective tissue in the muscles. In order to measure the total area of endo- and perimysial fibrosis digitally, microscopic images of whole longitudinal sections of the muscle were collected from the distal to the proximal tendon. Images were correlated and connected to obtain the whole longitudinal sections with the help of the Axio Vision program (release 4.4; Carl Zeiss, Göttingen, Germany). These compound pictures were then edited with an image analysis system (KS 400 3.0; Carl Zeiss). Tendinous structures were removed by the investigator and the absolute area of red connective tissue was measured and normalized to the total muscle area.

### Statistics

The arithmetic mean and SD were determined for each measure. Analysis of statistical significance was performed using the non-parametric Wilcoxon test for dependent samples when comparing measures intra-individually, and the non-parametric Mann-Whitney U test was used for independent values. The level of significance was set at 0.05.

## Results

### Mechanical testing

The maximally reached twitch contraction force remained almost constant with differences of less than 5% after 5 consecutive stimulations. Control muscles without trauma had a mean fast-twitch contraction force of 0.85 (SD 0.22) N.

Stimulation of the sciatic nerve over a second yielded tetanic contractions in all the muscles tested, resulting in a maximum contraction force of 1.49 (SD 0.32) N in the control muscles, which amounted to 175% of the value after fast-twitch stimulation (p < 0.001).

**Figure 2.**
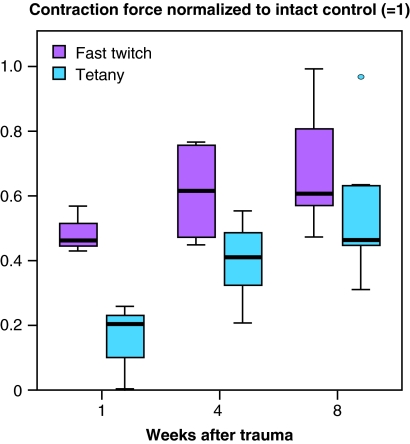
Time course of muscle function. Contraction forces were normalized within individuals to the forces of uninjured control muscles.

During the evaluated regeneration period, a continuous increase in contraction force after fast-twitch stimulation and after tetanic stimulation could be observed, describing the functional regeneration of the traumatized soleus muscle over time (Figure 2). One week after crush trauma, the mean contraction forces after tetanic stimulation amounted to 0.34 (SD 0.05) N. After fast-twitch stimulation, almost the same maximum force (0.32 (SD 0.04) N) was reached, indicating that the injured muscles had no possibility to generate additional power through activation of reserve fibers at that time.

Normalization against the right soleus muscle, which served as an internal control, showed a lag of regeneration of tetanic compared to twitch contraction force of 27% of the force generated by the healthy muscles 1 week after trauma (fast twitch 49% (SD 7), tetany 23% (SD 4) of the control side, p < 0.001). This lag of the tetanic contraction forces decreased over time, with the tetanic being 21% and 13% less than the normalized twitch contraction forces at weeks 4 and 8 after crush injury (week 4: fast twitch 61% (SD 13) of the control side, tetany 40% (SD 12), p = 0.028; week 8: fast twitch 68% (SD 19) of the control side, tetany 55% (SD 13), p = 0.028). This could be described by the correlation between the twitch/tetanus ratio and time after trauma (weeks), which showed a coefficient of correlation of 0.79 (p < 0.001).

### MRI findings

**Figure 3.**
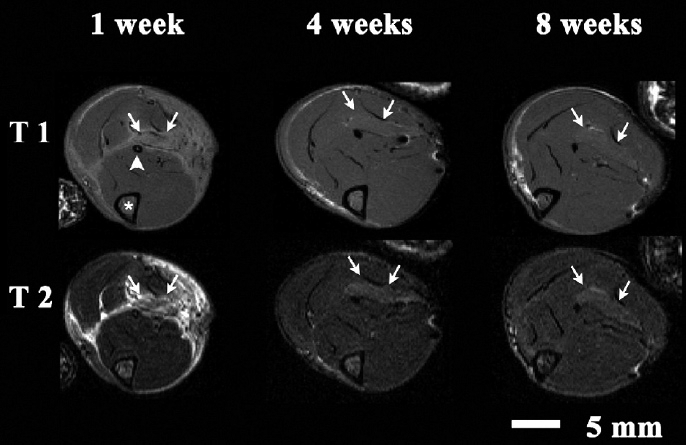
Time course of T1 and T2 signals after crushing of soleus muscle. Axial scans of the left lower leg of rat 2 in the MRI group, 1, 4, and 8 weeks after trauma. The asterisk shows the tibia and the arrowhead shows the fibula. Arrows: soleus muscle.

Sequential MRI measurements showed a continuous time course of soleus muscle morphology. 1 week after trauma, T1 and T2 scans demonstrated a clear contrast between the injured muscles and the surrounding superficial and deep flexors, the latter presenting with low signal intensity (Figure 3). The signals of the soleus muscles showed pronounced inhomogeneities in both scan modes. The alterations observed affected the whole muscle tissue. In the T2-weighted images, interstitial edema could be detected within the soleus fascia and at the site of the approach. A posttraumatic seroma was located between the soleus and the gastrocnemius muscles.

4 weeks after injury, these seroma and the edematous changes within the fascia had been resorbed to a great extent. The elevated signal intensity of the soleus muscles had decreased, but there was still a clear contrast between the injured muscles and their surroundings. Also, the inhomogeneities within the traumatized muscle tissue had decreased.

8 weeks after injury, the soleus muscles were still slightly elevated in their signal intensity in the T2-weighted scans, whereas in the T1-weighted scans the signal had almost adapted to the surrounding muscle tissue. The signal elevation was homogenous.

**Figure 4.**
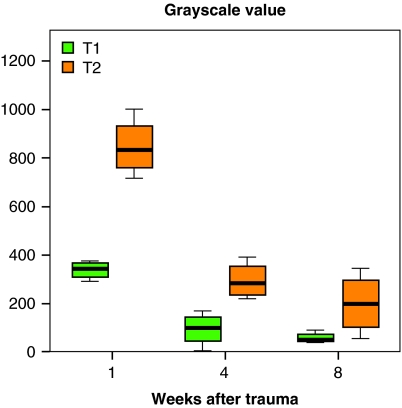
Time course of the differences in the grayscale values of soleus muscles and uninjured anterior tibial muscles 1, 4, and 8 weeks after soleus trauma in T1- and T2-weighted sequences. P-values: 1 week vs. 4 weeks, T1 + T2: p < 0.001; 1 week vs. 8 weeks, T1 + T2: p < 0.001; 4 weeks vs. 8 weeks, T1 and T2: p = 0.22 (ns) and p = 0.02, respectively.

The mean signal elevations of the injured soleus muscles in the T2-weighted scans were quantified at each time point, as shown in Figure 4. A clear time course of an exponential decrease in the grayscale values over time could be discerned, with the differences between traumatized and healthy muscle tissue being only minimal for T1 signals 8 weeks after injury.

### Descriptive histology

**Figure 5.**
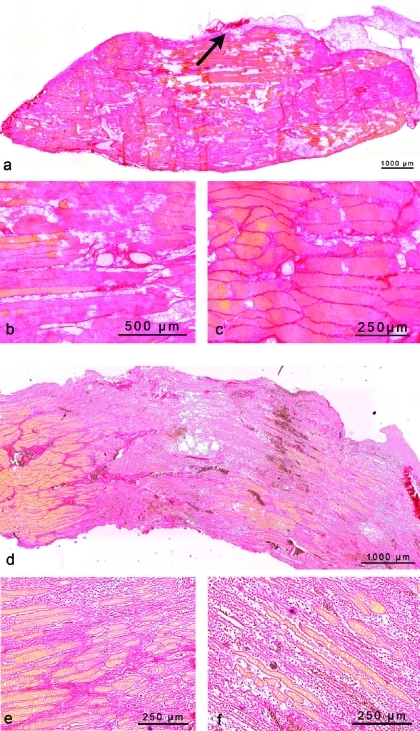
Longitudinal cryo-sections of crush-injured soleus muscles stained with Picrosirius red. a. Overview showing the angular uninjured region at the middle of the section (from day 1 after injury). The insertion of the neurovascular bundle is indicated by an arrow. b. Detail of panel a showing ruptured myofibers as evidence of shearing-type injury. c. Detail of panel a showing swollen, prenecrotic myofibers as evidence of in situ necrosis happening in parallel to the latter type of injury. d. Overview of the uninjured zone and the distal crush zone of a soleus muscle from day 2 after crush injury. e. Detail of panel d showing the border zone of injured and uninjured muscle with an interstitial inflammatory reaction. f. Myofibers in the crush zone surrounded by inflammatory cells in loose connective tissue.

**Figure 6.**
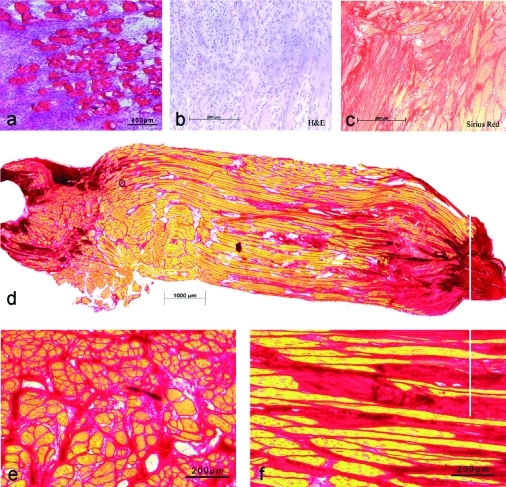
Histological staining of soleus muscles 4 days and 1, 4, and 8 weeks after crush injury. a. Cryo-section with HE staining of cross-sectioned myofibers surrounded by inflammatory cells on day 4 after injury. b. Paraffin section with HE staining of regenerating myofibers showing loose deposition of connective tissue with a high degree of cellularity and regenerating muscle fibers one week after trauma. c. Paraffin section with HE staining showing dense connective tissue with local scar formation and interstitial fibrosis 4 weeks after injury. d. Cryo-section with Sirius red staining: an overview of the injured soleus muscle 8 weeks after trauma showing typical interstitially pronounced fibrosis in the distal and proximal crush zone. e. Detail of panel d showing cross-sectioned myofibers surrounded by collagenous fibrotic tissue. f. Detail of panel d showing (longitudinally) sections of myofibers and interstitial fibrosis.

Sirius red-stained sections from the first day after injury showed red swollen, prenecrotic myofibers with intact boundaries on the one hand, and ruptured myofibers on the other, indicating the prevalence of the two main types of muscle injury: in-situ necrosis and shearing (Figure 5a). The fact that the basal laminae were injured in the latter case can be deduced from the myofiber ruptures initially observed (Figure 5b) and from the amount of isolated regenerating myofibers on the days studied thereafter (Figure 5d-f). What can also be observed in sections of the first and following days is the nature of the trauma—a global crush injury of the soleus muscle with a histologically obvious spared zone at the site of the neurovascular insertion (Figure 5a and d). The spared region was also affected by the trauma, as seen by the initial infiltration of inflammatory cells and interstitial hematoma, but this area showed a pronounced conservation of myofibers compared to the proximal and distal regions. This observation is shown in Figure 5 panels a, d and e, and can be interpreted according to the localization of the survival zone and regeneration zone after laceration trauma described in the work of Kääriäinen et al. (1998). The infiltration of inflammatory cells could be observed immediately after injury, showing a maximum on days 2 and 4 and remaining up to the end of the first week. At this time, most of the initial hematoma had already been removed and been replaced by loose connective tissue infiltrated mainly by monocytes and macrophages (Figure 6b). Centronucleated regenerating muscle fibers constituted the majority of the fibers at one week after trauma.

**Figure 7.**
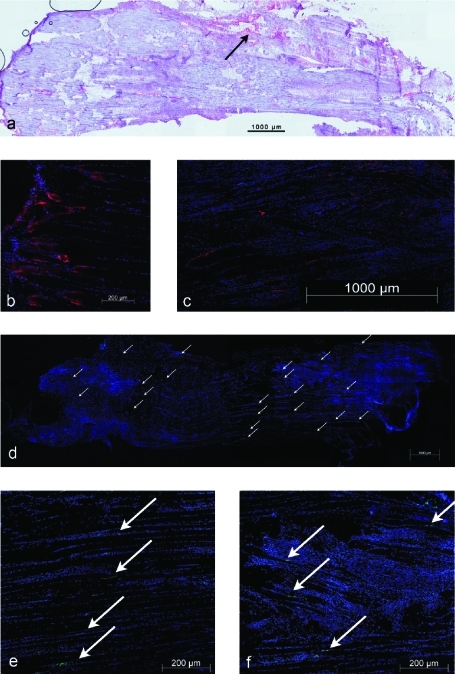
a. Factor VIII staining of a longitudinally sectioned soleus muscle 4 weeks after crush injury, showing the distribution of vessels in the injury zones. b. Cryo-section with an immunohistochemical stain for nestin in a healthy soleus muscle (counterstain: DAPI), myotendinous transition zone. c. Staining for nestin in a longitudinally sectioned soleus muscle (counterstain: DAPI). Nestin immunoreactivity was dispersed throughout the muscle and could be found not only at the tips of regenerating myofiber stumps but also at the lateral aspects of the fibers. d. Overview of a-bungarotoxin staining of a longitudinally sectioned soleus muscle (counterstain: DAPI). The white arrows indicate multiple, newly developed neural endplates in the proximal and distal crush zones of the muscle. e. Detail of panel d depicting a linear distribution of endplates in the uninjured region. f. Detail of panel d depicting endplates within the crushed area.

The distribution of newly formed blood vessels was uniform, indicating that there was a homogenous regeneration process in the crush zones, as can be seen in a representative section stained for factor VIII (Figure 7a). Vessels were detected at day 4 after trauma since the quick onset of the angiogenic process in skeletal muscle after trauma peaked within the first 5 days (Jarvinen 1976, Jarvinen et al. 1983).

The distribution of newly developed MTJs was also homogeneous and not confined to certain parts of the injured soleus. Figure 7b shows the myotendinous transition zone of a healthy soleus muscle, where immunoreactivity to nestin could be seen at the end zones of the soleus myofibers but there was no reactivity to a-bungarotoxin. In the crush-injured soleus muscles, nestin immunoreactivity was dispersed throughout the muscle and was not only found at the tips of regenerating myofiber stumps but also at the lateral aspects of the fibers, indicating connections with the interstitially developed collagenous tissue and participation of the fibers in the contraction process. Co-reactivity to a-bungarotoxin was observed in only a few cases (Figure 7c). Figure 7d-f shows the a-bungarotoxin-stained neural endplates 4 weeks after injury, which could be detected on myofibers disseminated in the crushed areas of the muscle. We also observed this 8 weeks after injury. Before injury, neural endplates could only be seen in a linear distribution in the region of the entry point of the neurovascular structures.

4 weeks after injury, inflammation had vanished and fibrotic tissue of a dense type had developed, although fibroblast nuclei still had a loose chromatin structure in many places, being a sign of activity. Apart from places of local scar formation, fibrotic tissue was evident especially in the interstitium between the myofibers (Figure 6c).

8 weeks after injury, maturation of connective tissue had already taken place, with spindle-shaped nuclei being the dominant fibroblast phenotype. Mature collagen fibers had replaced the loose reticular network to a large extent, as seen with polarization microscopy in slides stained with Sirius red. Few regenerating muscle fibers were still present. Mature muscle fibers were surrounded by an interstitial mesh of fibrotic tissue, representing endo- and perimysial collagen deposition (Figure 6d-f).

### Histomorphometry

Histomorphometric evaluation showed that 1 week after severe crush injury, 40% of the muscles were occupied by collagenous connective tissue, representing the loose, still active tissue described above. After 4 weeks, this area was reduced to 24% (p = 0.05) and it was still at this value at week 8 after trauma (Table).

**Table:** Relative amount of collagenous connective tissue over time referenced to the whole area of the muscle sections in percent (SD)

Weeks after trauma	1	2	3
Area of collagenous connective tissue	40.4% (3.5)	23.5% (7.1)	25.4% (7.3)

## Discussion

Experimental research on skeletal muscle trauma requires reliable animal models. These should afford the possibility of studying new therapeutic methods, especially with regard to their influence on functional and microstructural regeneration.

We used a modified standardized blunt trauma of the rat soleus muscle to establish a model of a selective injury. The regeneration process of traumatized skeletal muscle tissue could therefore be analyzed and described over time from a mechanical, histological, and MR-morphological point of view. In contrast to previously described whole soleus crush injuries (Bassaglia and Gautron 1995, Fink et al. 2003), the area of the neuromuscular junctions in the soleus muscle, a well-defined region at the midbelly of the muscle, was spared in order to create a model of a trauma selectively affecting the muscle fibers and the interstitial tissue without harming the main innervation of the organ. We therefore avoided an initial complete denervation, which would have made it impossible to differentiate between the consequences of denervation and of myofiber trauma. The advantage of the trauma model presented here is that with the greatest degree of possible myofiber injury without denervation injury, the effects of new therapies for skeletal muscle trauma can be analyzed with the lowest bias in the intrinsic regeneration capacities of the muscle itself with the maximum range for a possible therapeutic effect.

Mechanical evaluation showed a tetanic contraction force—describing the capacity of force development of a muscle—of 1.5 N in the uninjured soleus muscles, which is in accordance with the literature (Anderson et al. 1999). In contrast to other settings (Meffert et al. 2008), the force measurement protocol used in this study allowed us to analyze the function of a single muscle without any interference from other musculature. 1 week after severe crush injury, contraction forces in both stimulation modes had almost the same values (tetany 0.3 N, fast twitch 0.3 N). Normalization of the forces to the uninjured internal control muscle (= 1.0) demonstrated that the injury had its greatest functional impact on tetanic contractions with a reduction to 0.23, while twitch contraction remained at almost half of the value of the healthy muscles after the first week. Two conclusions can be made from these findings: (1) facilitation of muscle force development was not possible for the damaged fibers, and (2) additional reserve fibers could not be recruited during repetitive stimulation of the muscle.

Facilitation is an elementary phenomenon when muscle fibers are prompted with successive electrical stimuli. A second stimulus results in a markedly increased peak force when given at a short interval (optimally, 1.3–1.4 times the duration of the previous twitch) (Parmiggiani and Stein 1981). A prolongation and increase in local Ca^2+^ influx (Endo 1977) and increased muscle stiffness (Rubinstein et al. 1998) after the first twitch are thought to be responsible for the mechanism, both being properties of the muscle that were altered by the injury.

In our model, both types of skeletal muscle injury—in situ necrosis and shearing—co-exist. The latter leads to the separation of myofibers, which results in split regenerating fibers (as also described by Schmalbruch (1976) after whole muscle crush and by äärimaa et al. (2004) after transection injury). These myofibers have no contact with the neuromuscular junctions at the mid-belly of the soleus muscle and need to re-establish their contact with the nervous system. This could be demonstrated in our model by the development of new neuromuscular endplates with a homogenous distribution within the crush area. Due to the character of the trauma (lack of a linear fibrotic border), we cannot deduce whether the nerve outgrowths pierced through fibrous tissue or whether they went along only mildly altered interstitial paths. The extent of the trauma and the amount of fibrosis indicate, however, that at least part had to establish contact via scar tissue.

Posttraumatic soleus microstructure is dominated by the developing fibrosis. After transection or segmental crush injury, fibrosis is confined to a small area. The regenerating myofibers then develop new myotendinous junctions within this dense connective tissue (Hurme and Kalimo 1992), allowing the muscle almost complete functional regeneration (Kaariainen et al. 1998, Menetrey et al. 1999), provided the distal part of the affected muscle has not been severed from the nerve supply (Garrett et al. 1984). The establishment of new myotendinous junctions of the regenerating myofibers with the surrounding fibrotic interstitium together with the development of new neuromuscular endplates shows that the fibers again participate in the contraction process after regeneration. The mechanical evaluation described the functional regeneration of the muscle, with an increase in fast-twitch strength to two-thirds of that in the uninjured control muscle at 8 weeks after trauma, being only 13% higher than the normalized tetanic strength. This shows the recovery of the chemical and mechanical properties of the soleus muscle over time.

However, contraction forces at 8 weeks in our model lacked half (tetany) and one third (fast twitch) of the forces of the uninjured muscle. Histological evaluation at this time showed that there was pronounced interstitial fibrosis, which differs from the local fibrosis described above. It can be deduced that not only contractile properties but also passive mechanical properties such as muscle stiffness are altered by this tight collagenous network, also affecting force development via reduced compliance of the muscle. Together with the replacement of contractile muscle substance with fibrotic tissue, this reduction in compliance can still be responsible for the residual lag of contraction forces 8 weeks after trauma. Although the character of the collagenous tissue changes, the amount appears to stay at a constant level of 25% of the muscle tissue. Regenerative therapies for supported muscle healing should take this into account, since not addressing fibrosis means not being able to fully reconstitute muscle forces after injury.

The increase in contraction forces during the observation period was accompanied by a decrease in T1 and T2 hyperintensity in the MR measurements. The time course of this development described the decrease in the acuity of the trauma. Morphologically, MR scans showed a disorganized aspect of the muscle tissue one week after trauma with an inhomogeneity of voxel signals, which could only be observed to a small degree 4 weeks after trauma. This development corresponded well with the histological sections showing short regenerating muscle fibers separated by loose connective tissue at the first time investigated, and showing successive reorganization 4 and 8 weeks after trauma. Fibrotic tissue exhibits low signal intensity on all pulse sequences (Elsayes et al. 2006). 8 weeks after injury, the structure of the soleus muscles appeared to be homogenous and could hardly be differentiated from the surrounding muscles. This demonstrates that the interstitial fibrosis, which is one of the main problems for the contractile function, cannot be detected by standard MRI sequences, even when high field scanners are used.

We believe that our model will be of value for studying the influence of therapeutic approaches at several different levels of skeletal muscle regeneration.
